# Efficient and Dynamically Consistent Joint Torque Estimation for Wearable Neurotechnology via Knowledge Distillation

**DOI:** 10.3390/bioengineering13040474

**Published:** 2026-04-17

**Authors:** Shu Xu, Zheng Chang, Zenghui Ding, Xianjun Yang, Tao Wang, Dezhang Xu

**Affiliations:** 1 Science Island Branch, Graduate School of USTC, University of Science and Technology of China, Hefei 230026, China; shuxu@mail.ustc.edu.cn (S.X.);; 2 Institute of Intelligent Machines, Hefei Institutes of Physical Science, Chinese Academy of Sciences, Hefei 230031, China; 3 School of Artificial Intelligence, Hainan Normal University, Haikou 571127, China; 4 School of Artificial Intelligence, Anhui Polytechnic University, Wuhu 241000, China; xdz@ahpu.edu.cn

**Keywords:** wearable neurotechnology, joint torque estimation, knowledge distillation, physics-guided machine learning, inertial measurement unit (IMU), on-device inference, motor rehabilitation

## Abstract

Wearable neurotechnology depends critically on continuous movement monitoring to characterize motor impairment and recovery in real-world settings. While joint torque serves as a clinically essential kinetic marker, estimating it directly on-device from inertial signals remains challenging due to stringent computational, memory, and energy constraints. Lightweight pipelines typically omit computationally expensive time–frequency processing; however, this omission degrades the observability of dynamics encoded in 1D IMU signals and diminishes the effectiveness of standard knowledge distillation strategies. To enable reliable on-device torque inference, we propose a Physically Guided Dual-Consistency Knowledge Distillation (PDC-KD) framework that explicitly integrates biomechanical priors into the learning process through two collaborative pathways: parameter-manifold alignment and physics-guided compensation. The student network receives guidance through Fisher-information-weighted parameter transfer, ensuring robust knowledge distillation despite significant model capacity mismatch. Furthermore, the framework incorporates a physics-guided regularization term that enforces dynamically consistent torque trajectories via a numerically stable Cholesky-parameterized constraint. Experiments demonstrate that the student model preserves teacher-level predictive accuracy while operating within the stringent resource constraints of edge devices (achieving a 98% parameter reduction, ∼2× faster inference, and ∼1 ms latency). Moreover, the proposed method yields torque estimates with enhanced dynamical consistency, providing an efficient biosignal-processing solution for wearable neurotechnology platforms demanding real-time movement analytics.

## 1. Introduction

Real-time monitoring of human joint torque provides critical quantitative feedback for intelligent rehabilitation assessment, biomechanical analysis, and exoskeleton control systems [1,2]. However, these systems are typically deployed on embedded platforms with limited computational capacity, stringent power constraints, and demanding real-time requirements. Consequently, high-fidelity deep learning models face substantial deployment bottlenecks [3]. For example, mainstream time–frequency approaches employ the continuous wavelet transform (CWT) to construct two-dimensional representations that enhance feature extraction accuracy. However, the associated preprocessing is computationally intensive, increasing single-step inference latency to over 150 ms [4], thereby limiting real-time interaction on wearable devices. Under stringent hardware constraints, edge deployment often removes such preprocessing and reverts the input modality from a two-dimensional time–frequency spectrum to a one-dimensional time-series signal.

This transformation is not merely a linear dimensionality reduction but represents structural degradation of the observation space [5,6]. Under such degradation, local energy distributions and spectral evolution patterns that were explicitly encoded in the time–frequency plane become implicitly coupled due to reduced observability [7,8]. This results in a pronounced observability gap between teacher and student models. The reduced shared representational basis across heterogeneous architectures undermines the feature-manifold isomorphism assumptions underlying conventional knowledge transfer methods [9,10]. Consequently, in the absence of well-aligned intermediate representations, establishing effective mapping relationships between architectures with unequal observability becomes a central challenge for cross-model knowledge distillation.

Owing to the observability gap and the reduced shared representational basis, existing knowledge distillation methods exhibit limited applicability in heterogeneous settings. Mainstream feature-alignment approaches assume the existence of a mappable isomorphic manifold between teacher and student feature spaces [11,12]. Under structural degradation of the input modality, however, this assumption is often violated, and enforced feature projections may produce unstable or non-physical mappings. Response-level distillation is further constrained by the low-rank nature of regression outputs, which limits its ability to convey the high-dimensional inference structure of the teacher model. Such approaches rely primarily on end-to-end numerical fitting and lack structural guidance, making it challenging to correct deviations from physical consistency caused by incomplete observations [13,14]. Traditional physics-based constraints are typically implemented as posterior regularization terms that primarily restrict the solution space boundaries [15]. When key time–frequency information is systematically absent at the input stage, such boundary constraints cannot substitute for the missing observational structure.

Therefore, under heterogeneous settings with incomplete observability, two challenges persist: constructing a representation-independent knowledge transfer pathway and incorporating deterministic exogenous mechanisms to supplement purely statistical inference. In this context, the central difficulty extends beyond specific algorithmic designs to identifying the appropriate level of information that can serve as a stable knowledge carrier. When the input modality degrades from two-dimensional time–frequency representations to one-dimensional time-series signals, the student model loses explicit spectral information and the shared basis required for reliable feature alignment. Consequently, representation-based alignment strategies become insufficient.

Instead of enforcing unstable feature-level mappings, the proposed approach leverages (1) parameter-level structural geometry to inherit the inference topology of the teacher model and (2) physics-based dynamical priors to compensate for dynamical information lost due to dimensionality reduction. By combining structural inheritance with physics-guided compensation, a Physically Guided Dual-Consistency Knowledge Distillation (PDC-KD) framework is proposed to enable reliable cross-model knowledge transfer under constrained edge-computing resources and simplified input modalities.

The main contributions are summarized as follows:

1. A transfer strategy based on parameter-manifold alignment is proposed to replace conventional feature-level alignment. To address intermediate feature mismatch across heterogeneous architectures, a shared anchor space is constructed to enable student models to inherit the teacher’s inference topology at the parameter level, thereby reducing reliance on feature-space isomorphism.
2. A physics-guided exogenous information compensation mechanism is established. Unlike conventional boundary regularization strategies, implicit biomechanical priors are incorporated as an independent exogenous information source. Robust physical operators are used to compensate for the loss of dynamical consistency resulting from input dimensionality reduction.
3. The effectiveness of the proposed framework for lightweight edge deployment is validated. Experiments on a standard IMU-based dynamic regression task demonstrate that the framework reduces model parameters by approximately 98% while maintaining predictive performance comparable to a high-fidelity teacher model, confirming its practical applicability in resource-constrained engineering scenarios.

The remainder of this paper is organized as follows. Section 2 reviews related work on wearable dynamics estimation and heterogeneous knowledge distillation, highlighting current limitations. Section 3 details the PDC-KD framework, including the shared anchor-space parameter-manifold alignment strategy and the physics-guided dynamical compensation mechanism. Section 4 presents the experimental setup and evaluates prediction accuracy, computational efficiency, and physical consistency on a standard lower-limb movement dataset, followed by ablation and baseline comparisons. Section 5 discusses the effective boundary of structured knowledge transfer and analyzes the influence of physical priors, clarifying the limitations of the proposed method. Section 6 concludes the paper and outlines directions for future research.

## 2. Related Work

### 2.1. Dynamics Characterization: From Physics-Driven to Data-Driven

**Figure 1:**
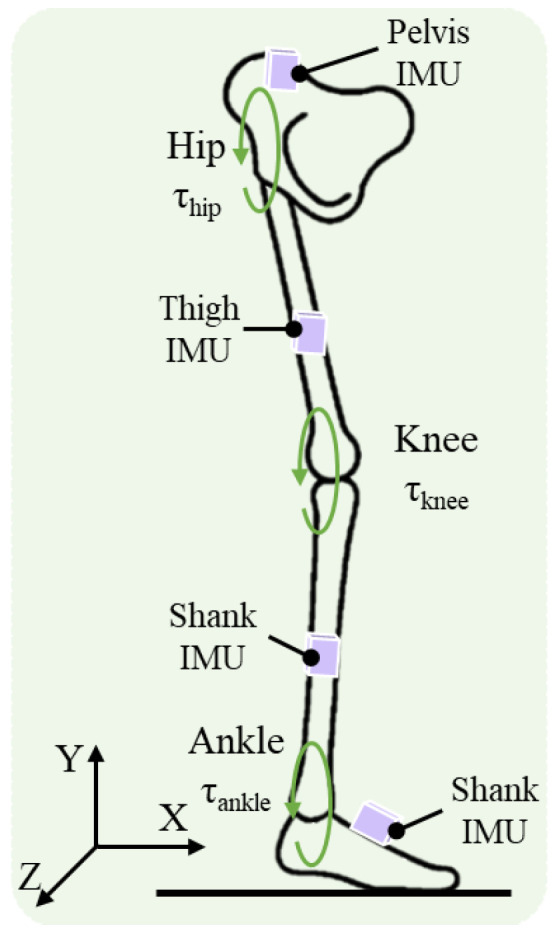
Schematic illustration of the wearable IMU sensor placement and target joint torques for lower-limb dynamics estimation. Four IMU sensors are attached to the pelvis, thigh, shank, and foot segments. The three target joints—hip, knee, and ankle—are indicated by green circular arrows representing the estimated joint torques $\tau_{\mathrm{hip}}$, $\tau_{\mathrm{knee}}$, and $\tau_{\mathrm{ankle}}$, respectively.

Wearable inertial measurement unit (IMU)-based human dynamics estimation has attracted sustained research interest in motion analysis and rehabilitation engineering, as illustrated in Figure 1. Existing approaches can be broadly categorized into physics-driven modeling and data-driven learning. Physics-driven methods are grounded in classical rigid-body dynamics. They construct articulated human models and employ Newton–Euler or Lagrangian formulations to analytically compute joint torques from sensor-derived kinematic parameters [1]. Under controlled laboratory conditions or when inertial parameters are known, these methods establish explicit causal relationships and provide a theoretical basis for biomechanical analysis.

Data-driven methods leverage large-scale motion datasets to learn end-to-end mappings from raw IMU signals to joint torques, typically using deep neural networks to capture nonlinear representations [4,16]. In standard gait or periodic motion scenarios, these models demonstrate strong functional approximation capabilities and adaptability in multimodal fusion and cross-condition estimation. To bridge physics-driven and data-driven modeling, recent studies have incorporated biomechanical principles into neural network architectures. These approaches introduce physics-informed constraints via loss functions [17] or embed anatomically informed inductive biases, balancing data-fitting flexibility with physically grounded regularization.

As application scenarios expand from controlled laboratory settings to real-world daily environments, observation conditions for wearable dynamics estimation become increasingly complex. In free-movement conditions, sensors are affected by soft-tissue artifacts, sensor-to-segment misalignment, and environmental noise [18,19]. Moreover, inter-individual biomechanical variability and diverse movement patterns impose stringent requirements on model generalization and long-term stability. Existing studies indicate that, in the presence of non-stationary disturbances, approaches relying solely on statistical feature learning struggle to maintain robustness in cross-subject and long-term monitoring tasks without structural constraints [20,21]. To address these challenges, prior work has introduced biomechanical constraint terms to impose physical boundaries [17] or employed deep time–frequency representations to enhance robustness against complex signal interference [4]. Such strategies aim to mitigate estimation fluctuations under non-ideal deployment conditions and improve the reliability of dynamic monitoring in interactive environments.

Beyond IMU-based methods, vision-based skeleton tracking systems such as Microsoft Kinect have also been explored for human motion analysis and biomechanical estimation. Esmaeeli et al. demonstrated that skeletal joint data extracted from Kinect can be used for exercise recognition and exercise quality assessment, highlighting the potential of depth-sensor-derived kinematics for rehabilitation monitoring [22]. Furthermore, Plantard et al. investigated the feasibility of computing joint torques via inverse dynamics using occlusion-corrected Kinect data, showing that markerless depth cameras can provide reliable torque estimates even under partial occlusion [23]. These studies indicate that skeleton tracking offers a complementary sensing modality; however, its applicability in unconstrained real-world environments remains limited by occlusion sensitivity and the absence of fine-grained inertial information. More broadly, the growing demand for on-device wearable signal processing extends beyond gait dynamics to diverse neurotechnology applications, including motor symptom monitoring in neurological disorders [24], further motivating the development of computationally efficient IMU-based approaches for real-world deployment.

### 2.2. Heterogeneous Knowledge Distillation and Modality Mismatch

In highly heterogeneous or cross-modal scenarios, where significant mismatches exist in input modalities, input modalities, information dimensionality, or feature-distribution characteristics between teacher and student models, existing knowledge distillation methods are constrained by their underlying mathematical assumptions. Feature-level distillation is theoretically sound in isomorphic or weakly heterogeneous tasks; however, its effectiveness depends on the mappability and semantic consistency of intermediate representations [25]. Such methods employ learnable projection operators to establish mappings between teacher and student feature spaces for representation alignment. However, when the input modality transitions from a two-dimensional time–frequency representation to a one-dimensional time-series signal, this mapping assumption becomes difficult to satisfy [4,7]. Linear or nonlinear projection layers primarily perform feature transformation or compression and cannot reconstruct explicit time–frequency structures that are systematically absent at the input stage. Forcing alignment between heterogeneous features with substantially different information densities may induce unstable or non-physical mappings, thereby limiting the effectiveness of cross-modal knowledge transfer [11].

To address the limitations of feature alignment, response-level distillation has been proposed to bypass intermediate structural discrepancies. However, the supervisory information structure in regression tasks differs fundamentally from that in classification-based distillation. In classification settings, knowledge distillation leverages the teacher’s soft probability distribution to encode structured information through inter-class relationships. In contrast, regression tasks such as joint torque estimation produce unbounded continuous outputs, limiting their capacity to convey structured relational information. In highly dynamic nonlinear modeling scenarios, compressing the teacher’s high-dimensional inference structure into low-dimensional continuous outputs may induce prediction smoothing, reduce sensitivity to high-frequency weak features, and ultimately impair generalization under complex conditions [13,14].

Relational and manifold distillation methods move beyond pointwisefeature alignment and instead emphasize preserving geometric relationships within the feature space or among samples [12,26,27]. These approaches assume that relative sample positions or manifold topology remain transferable across heterogeneous architectures. However, they still rely on the preservation of activation distributions to maintain topological similarity. When input modality changes alter the feature-generation mechanism, the assumption of topological consistency may no longer hold. In extreme heterogeneous scenarios, changes in observation dimensionality reshape the feature-manifold geometry, thereby limiting the effectiveness of activation-distribution alignment.

In scenarios characterized by extreme heterogeneity and cross-modal distillation, existing methods encounter fundamental technical barriers when confronted with significant mismatches in observational information. Although current strategies perform effectively in isomorphic compression settings, addressing heterogeneous cross-modal scenarios requires the development of robust knowledge carriers and transfer mechanisms that do not rely on input-space similarity, particularly when physical isomorphism between input modalities is absent.

### 2.3. Structural Alignment and Physics-Guided Learning

Beyond aligning intermediate features or sample relationships, recent knowledge distillation research has explored transfer mechanisms at the level of model parameters and weight structures. These approaches constrain similarities in parameter distributions or structural configurations between teacher and student models, establishing connections beyond feature activations. Representative strategies include aligning statistical moments of weights, preserving inter-parameter correlation structures, and leveraging local geometric properties of the parameter space to guide knowledge transfer. In scenarios involving substantial differences in input modalities that disrupt feature distribution consistency, parameter-based approaches rely on relatively stable structural properties of model weights and offer an alternative perspective for cross-architecture knowledge transfer. Distillation strategies have progressively shifted their alignment targets from dynamic feature responses to more stable parameter structures to preserve structural consistency across models under varying task constraints. In parallel, studies have explored intermediate adapter designs, information-flow modeling, and contrastive learning frameworks as complementary strategies to mitigate representational discrepancies across heterogeneous architectures [9,10,28].

From the perspective of information hierarchy selection, intermediate-representation alignment and parameter-structure alignment represent distinct research paradigms. The former emphasizes capturing input-driven activation behaviors and achieves knowledge transfer by aligning intermediate responses, whereas the latter concentrates on the structural organization and geometric properties of model parameters. Prior studies have shown that neural networks trained on similar tasks exhibit structured geometric distributions within their parameter spaces [29,30]. Inspired by optimization theory and continual learning, metric tools such as the Fisher information matrix have been used to characterize the local sensitivity of parameters to task objectives [31]. These perspectives analyze knowledge transfer from dynamic activation and static structural viewpoints, each offering complementary insights under distinct assumptions. Investigations into hierarchical parameter structures reflect an essential extension of distillation research in heterogeneous settings.

In parallel, data-driven modeling studies have incorporated physical priors or dynamical models to introduce external information into statistical learning frameworks. These studies embed physical laws as auxiliary constraints during training, for example, through regularization terms, boundary-condition enforcement, or posterior correction strategies based on known dynamical equations [32,33]. In addition, related efforts have incorporated physical constraints into student training to integrate exogenous biomechanical assumptions into the learning process [34].

## 3. Methodology

**Figure 2:**
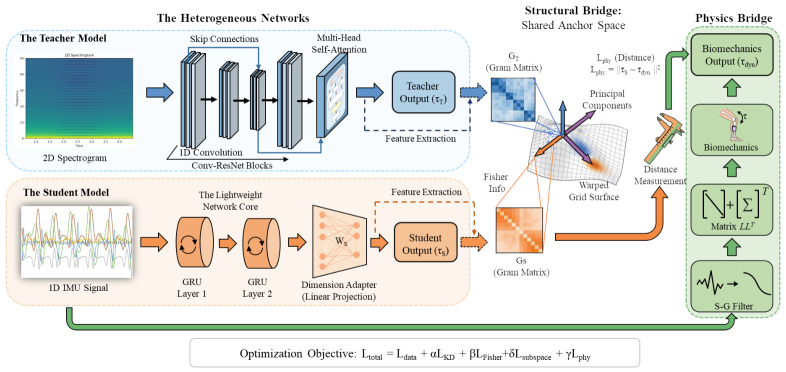
Overview of the proposed PDC-KD framework. The framework targets heterogeneous nonlinear dynamics estimation and comprises two collaborative learning pathways. The first pathway implements parameter-manifold alignment by establishing a shared anchor space via a dimensional adapter. Within this anchor space, Fisher information weighting and low-rank subspace constraints are integrated to achieve geometric alignment between the teacher and student parameter manifolds ($W_{T}$ and $W_{S}$), enabling inheritance of the teacher’s inference structure at the output-mapping layer. The second pathway introduces physics-guided learning, where robust dynamical operators construct a physical-consistency loss and embed Newton–Euler constraints into the training process, enhancing dynamical plausibility and prediction stability under representation degradation. In the 1D IMU Signal and 2D Spectrogram panels, different colored lines represent signals from distinct sensor channels (x-, y-, and z-axes).

Under edge-computing constraints, limited computational resources necessitate a simplification of the input modality from two-dimensional time–frequency spectra to one-dimensional time-series signals. This modality simplification introduces substantial architectural heterogeneity between teacher and student architectures. To address this challenge, a Physics-Guided Dual-Consistency Knowledge Distillation (PDC-KD) framework is proposed. The framework establishes a hierarchical optimization strategy that integrates parameter-manifold alignment with physics-guided information compensation (Figure 2). This design enables knowledge transfer and dynamical information reconstruction in lightweight deployment scenarios characterized by incomplete observability.

### 3.1. Problem Formulation and the PDC-KD Framework

The effectiveness of the PDC-KD framework is grounded in three theoretical premises that define its applicability:

1. **Task-consistency assumption:** The teacher and student models share an identical optimization objective and physical constraints within the same dynamic regression task.
2. **Parameter-proxy assumption:** When heterogeneous architectures hinder feature-space alignment, the structural configuration of weights in projection and adaptation layers is assumed to encode inference logic rather than merely support representation transfer.
3. **Computational-mediation assumption:** The shared anchor space serves as a mathematical reference for quantifying geometric relationships among heterogeneous parameter matrices, independent of intermediate feature representations or latent semantic spaces.

The proposed framework applies to regression tasks characterized by substantial structural differences in input modalities and well-defined dynamical priors. Its primary objective is to mitigate performance degradation in resource-constrained edge-computing environments, rather than to serve as a general-purpose alternative to isomorphic compression or conventional distillation.

The PDC-KD framework consists of two complementary pathways. The first pathway addresses the challenge of feature-level alignment under heterogeneous modality degradation by introducing a parameter-level structural alignment mechanism. Instead of transferring the entire encoder weights or network topology, it anchors the teacher’s projection layer ($W_{T}$) and the student’s dimensional adaptation layer ($W_{S}$), establishing a geometric correspondence within a shared anchor space. Fisher information weighting is combined with a low-rank subspace projection strategy to minimize structural discrepancies between their parameter manifolds. This mechanism enables the student model to approximate the teacher’s inference-boundary structure during output mapping, thereby reducing reliance on intermediate feature-space isomorphism.

The second pathway introduces physics-guided information compensation. Because input dimensionality reduction results in the loss of high-frequency information, physical priors are incorporated as additional sources of information. The framework constructs implicit inertial parameters and robust differential operators and imposes regularization constraints derived from the Newton–Euler equations. Rather than enforcing exact physical correctness of the outputs, this mechanism introduces data-independent inductive biases that suppress non-physical prediction oscillations induced by modality degradation and enhance robustness under practical engineering constraints.

### 3.2. Heterogeneous Architectures for Edge Deployment

A heterogeneous teacher–student distillation framework is adopted. The teacher model employs CWT to generate time–frequency representations and integrates convolutional networks with attention mechanisms to extract multi-scale dynamical features [4]. This architecture captures both local time–frequency structures and global correlations, thereby ensuring accurate torque estimation. However, its computational complexity and parameter scale limit deployment on resource-constrained edge devices.

The student model directly processes one-dimensional IMU time-series signals using a lightweight recurrent neural network to capture temporal dependencies, significantly reducing computational overhead. This simplification results in the systematic loss of frequency-domain structural information, thereby reducing observability and limiting the model’s representational capacity.

The structural disparity between the two networks renders their parameter-space geometries directly incomparable. To establish a computable structural correspondence, a linear dimensional adapter is appended to the student network, ensuring output dimensional consistency with the teacher’s projection layer. This design guarantees parameter dimensional compatibility, enables geometric comparability between heterogeneous weight matrices, and provides the basis for subsequent alignment within the shared anchor-space parameter manifolds. The core objective is to develop an effective and physically consistent knowledge transfer mechanism under heterogeneous conditions, rather than relying solely on conventional network-architecture optimization.

### 3.3. Path I: Parameter-Manifold Alignment via Shared Anchor Space

When the input modality degrades from a two-dimensional time–frequency spectrum to a one-dimensional time-series signal, the feature-generation mechanisms of the teacher and student models become substantially misaligned, rendering conventional feature-alignment distillation strategies ineffective. Accordingly, under the parameter-proxy assumption defined in the theoretical premises, a shared anchor space is constructed, and a parameter-level knowledge transfer pathway is introduced to replace conventional feature-alignment schemes.

#### 3.3.1. Construction of a Shared Parameter Anchor Space

Let $W_{T}$ and $W_{S}$ denote the projection weight matrices of the teacher and student models within the shared anchor space, respectively. Owing to the inherent difference in their original parameter-space dimensions ($D_{T}\neq D_{S}$), direct numerical approximation between them is not feasible. To address this dimensional inconsistency, the Gram matrix is introduced as a computational tool to characterize the second-order correlation structure of parameters in the anchor space [35], as formulated in Equation  (1).

(1) $$\begin{array}{cc} G_{T} & =\phi \left(W_{T}\right)=W_{T}^{⊤}W_{T},\\ G_{S} & =\phi \left(W_{S}\right)=W_{S}^{⊤}W_{S}, \end{array}$$

where $G_{T},G_{S}\in R^{D_{\mathrm{anchor}}\times D_{\mathrm{anchor}}}$. The Gram matrix transformation projects parameters of different original dimensions into a unified metric space without relying on input-level semantic representations. In this metric space, parameter alignment is formulated as a structural-consistency constraint on the corresponding Gram matrices.

This structural-consistency strategy alleviates the limitation imposed by asymmetric input observability, establishes a structural similarity metric across heterogeneous architectures, and formulates the corresponding alignment loss as shown in Equation (2).

(2) $$L_{\mathrm{align}}={∥G_{S}-G_{T}∥}_{F}^{2},$$

where ${∥\cdot ∥}_{F}$ denotes the Frobenius norm.

**Figure 3:**
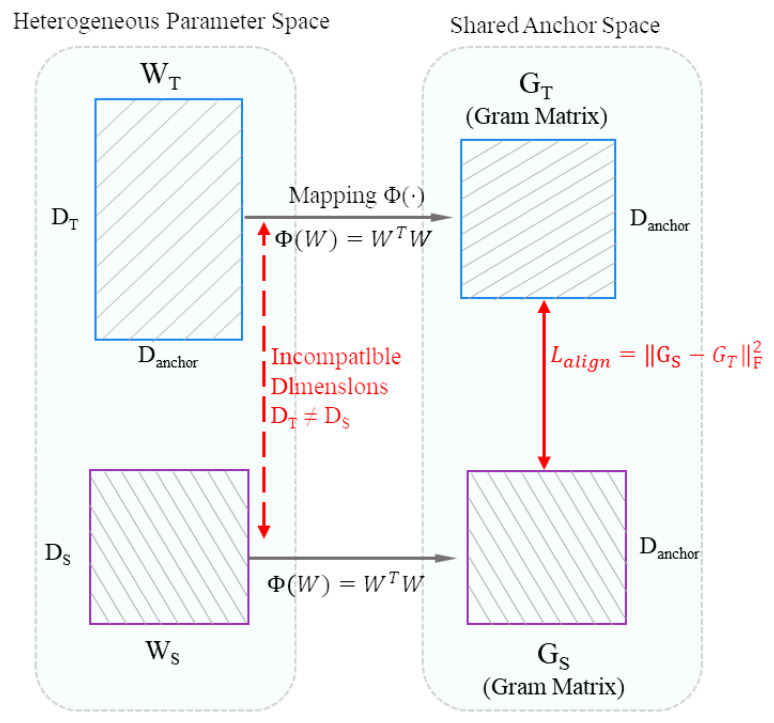
Anchor-space construction and parameter-manifold alignment. The (**left panel**) illustrates the incompatibility between heterogeneous weight matrices arising from a hidden-layer dimensional mismatch ($D_{T}\neq D_{S}$). The Gram matrix mapping Φ(·) projects heterogeneous parameters into a unified shared anchor space (**right panel**). This anchor space serves as a structural metric mediator, standardizing the dimensions of the Gram matrices ($G_{T}$, $G_{S}$) and minimizing structural discrepancies. The alignment loss $L_{\mathrm{align}}$ drives geometric consistency between the parameter distributions.

In Equation (2), the relationship between heterogeneous parameters is reformulated as a geometric correlation structure. By minimizing structural discrepancies between the Gram matrices, the student network is encouraged to approximate the teacher’s topological distribution on the parameter manifold. The parameter-manifold alignment mechanism alleviates the computational challenge posed by dimensional incompatibility between heterogeneous parameters and provides a mathematical basis for subsequent refinement via structural guidance (see Figure 3).

#### 3.3.2. Task-Sensitive Alignment via Fisher Geometry

Although the Gram matrix captures second-order correlations among parameters, it does not distinguish the relative contributions of individual feature dimensions to the final prediction. To compensate for the student model’s limited parameter capacity for handling high-dimensional features, Fisher information from the teacher network is introduced as a local sensitivity-weighting mechanism. Fisher information quantifies the second-order sensitivity of the loss function to parameter perturbations within the anchor space, and its approximate formulation is given in Equation (3).

(3) $$F_{T,kk}\approx E_{x∼D}\left[{\left(\frac{\partial L}{\partial z_{k}}\right)}^{2}\right]$$

Here, $z_{k}$ denotes the output variable corresponding to the *k*th dimension of the teacher network in the anchor space.

Fisher information characterizes the statistical sensitivity of model parameters to the task loss, independent of input-level feature saliency. The Fisher-based weighting mechanism eliminates the need for both input-sample comparability across heterogeneous models and semantic alignment of intermediate-layer features. While the anchor space provides a coordinate representation of parameters, Fisher information assigns a task-relevant local metric to this space, enabling differentiation of the relative importance of parameter directions.

Based on this sensitivity metric, the Fisher-weighted discrepancy between Gram matrices in the anchor space is computed to formulate a local sensitivity alignment loss, as defined in Equation (4).

(4) $$L_{\mathrm{Fisher}}=\sum_{k}F_{T,kk}{∥G_{S,k}-G_{T,k}∥}_{F}^{2}$$

The local sensitivity alignment loss elevates the distillation objective from purely geometric matching to task-aware structural alignment. The Fisher-weighting mechanism introduces a task-relevant local metric into the parameter manifold, approximating the directional distribution of prediction sensitivity within the parameter space. This mechanism guides size-constrained student models to prioritize alignment along structurally informative directions with higher information density.

#### 3.3.3. Principal Subspace Regularization

Fisher information constraints capture only local parameter sensitivity and do not reflect the global correlation structure within the anchor space. To complement this local perspective, a low-rank subspace alignment strategy is introduced to establish a structural regularization mechanism [36,37]. Specifically, singular value decomposition (SVD) is applied to the teacher’s Gram matrix $G_{T}$ to extract principal component subspaces and construct the corresponding projection operator $P_{T}$, as defined in Equation (5):

(5) $$P_{T}=U_{k}U_{k}^{⊤}$$

Here, $U_{k}$ contains the first *k* principal basis vectors derived from the teacher’s Gram matrix, forming the principal subspace of the anchor space.

**Figure 4:**
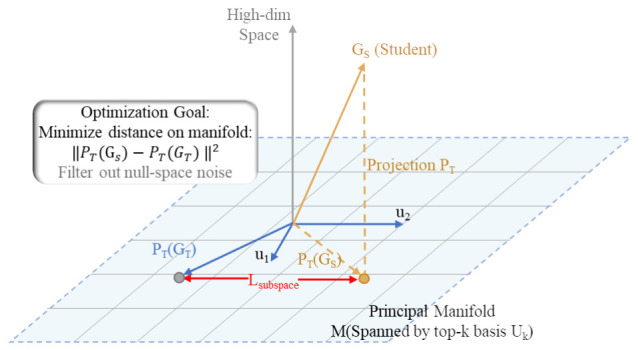
Geometry of low-rank subspace alignment. Illustration of the structural constraint mechanism for heterogeneous parameters within the shared anchor space. The principal components of the teacher’s Gram matrix span a low-rank manifold (the blue plane is defined by basis vectors $u_{1}$ and $u_{2}$). The student Gram matrix $G_{S}$ is projected onto this manifold using the projection operator $P_{T}$. The red double arrows denote the subspace alignment loss $L_{\mathrm{subspace}}$. The alignment mechanism minimizes the projection distance within the principal subspace, suppresses variations in minor components, and constrains the student network to follow the teacher’s dominant correlation patterns.

Under this projection operator, the student Gram matrix $G_{S}$ is projected onto the teacher’s principal subspace. The subspace alignment loss is defined as the structural distance after projection, as formulated in Equation (6), and its geometric interpretation is illustrated in Figure 4:

(6) $$L_{\mathrm{subspace}}={∥P_{T}G_{S}-P_{T}G_{T}∥}_{F}^{2}$$

This strategy effectively constrains the optimization trajectory of the student model parameters, prioritizing convergence along the principal directions defined in the teacher’s anchor space. By restricting the degrees of freedom within the low-rank subspace, this regularization term suppresses minor noise components during parameter optimization, promotes approximation of the teacher’s global correlation structure, and enhances the training stability of the lightweight student model.

### 3.4. Path II: Physics-Guided Compensation for Dynamical Consistency

As the input modality degrades from a two-dimensional time–frequency spectrum to a one-dimensional time-series signal, the student model experiences a systematic loss of frequency-domain structural information. This leads to observability degradation and induces high-frequency oscillations in the predictions that deviate from physical principles.

In contrast to Section 3.3, which emphasizes parameter-manifold distillation for inference-logic transfer, this section introduces an independent exogenous physical-compensation pathway. This physics-guided compensation mechanism extends beyond conventional solution-space regularization by incorporating human-body dynamical equations as an independent information source. It actively compensates for missing observational dimensions, restores dynamical-consistency features, and calibrates predictions at the signal level.

#### 3.4.1. Equivalent Inertia Modeling and Robust Operators

**Figure 5:**
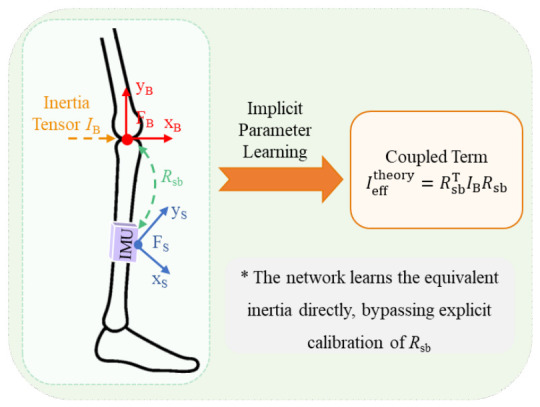
Coordinate mapping and parameter-coupling mechanism. The figure illustrates the geometric coupling between the human inertial tensor $I_{B}$ and the sensor orientation matrix $R_{\mathrm{sb}}$. The inertial parameters are integrated to form an equivalent inertial tensor $I_{\mathrm{eff}}$, which serves as a learnable compensation target. This formulation simplifies the decoupling and calibration of rotational transformations. The asterisk (*) indicates that the network learns the equivalent inertia $I_{\mathrm{eff}}$ directly during training, bypassing explicit calibration of $R_{\mathrm{sb}}$.

In practical wearable applications, calibrating the transformation between the sensor coordinate system and the human anatomical coordinate system remains a significant challenge. An equivalent-parameter modeling strategy is adopted to enhance engineering applicability. Specifically, the unknown coordinate transformation $R_{\mathrm{sb}}$ is incorporated into the equivalent inertial tensor $I_{\mathrm{eff}}$, which is treated as a learnable compensation variable and optimized automatically during network training. The resulting physical coupling relationship is formulated in Equation (7), as illustrated in Figure 5:

(7) $$I_{\mathrm{eff}}^{\mathrm{theory}}=R_{\mathrm{sb}}^{⊤}I_{B}R_{\mathrm{sb}}$$

Here, $I_{\mathrm{eff}}$ denotes the equivalent inertial tensor expressed in the sensor coordinate system; $R_{\mathrm{sb}}$ represents the rotation matrix of the sensor relative to the limb; and $I_{B}$ corresponds to the standard inertial tensor of the human body segment.

Based on the equivalent modeling framework, the Newton–Euler equations are used to evaluate the student model’s predictions. Owing to soft-tissue deformation and device-induced micro-motion artifacts, the dynamic equations serve as a physics-based approximation of rigid-body motion. They constrain the trend of joint torque variations rather than providing an exact analytical solution. The resulting dynamic-consistency relationship is formulated in Equation (8):

(8) $$\tau \approx I_{\mathrm{eff}}\dot{\omega }+\omega \times \left(I_{\mathrm{eff}}\omega \right)+G\left(\theta \right)$$

Here, τ (N · m/kg) denotes the joint torque; ω and $\dot{\omega }$ represent the angular velocity and angular acceleration, respectively; and G(θ) denotes the gravity compensation term.

#### 3.4.2. Physics-Consistent Residual Regularization

**Figure 6:**
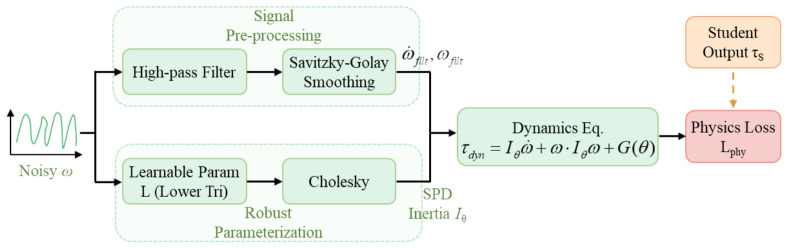
Computational flowchart of the robust physics-compensation mechanism. The upper branch applies a Savitzky–Golay filter to suppress noise in angular-velocity differentiation. The lower branch constructs a symmetric positive-definite (SPD) inertial tensor via Cholesky decomposition. The two mechanisms jointly operate within the dynamic equations to mitigate kinematic distortions induced by input simplification and enhance the numerical stability of gradient-based compensation.

To ensure stable gradient propagation in the physics-compensation pathway under noisy IMU measurements, both numerical stability and algebraic validity must be considered. As illustrated in Figure 6, a robust dynamical operator is introduced that integrates two core mechanisms.

First, a Savitzky–Golay smoothing differentiator is applied as a signal-preprocessing step to suppress high-frequency noise in the angular-velocity measurements. Second, a Cholesky-based reparameterization of the inertial parameter matrix is employed to enforce the symmetric positive-definite (SPD) property of the equivalent inertial tensor, thereby constraining the learnable parameters to remain within the physically feasible domain.

Based on the robust dynamical operator, a physics-compensation loss $L_{\mathrm{phy}}$ is formulated according to the Newton–Euler equations, as defined in Equation (9):

(9) $$L_{\mathrm{phy}}={∥\tau_{S}-\left(I_{\mathrm{eff}}{\dot{\omega }}_{\mathrm{filt}}+\omega_{\mathrm{filt}}\times \left(I_{\mathrm{eff}}\omega_{\mathrm{filt}}\right)+G\left(\theta \right)\right)∥}_{2}^{2}$$

The physics-compensation loss serves not only as a regularization term but also as a core structural guidance signal. By minimizing the physical residual, the loss encourages the student model to move beyond purely data-driven statistical regularities and actively recover dynamical trend information lost due to reduced perceptual dimensionality.

Implementation details of the robust operator and its stability analysis are provided in Appendix A, while the derivation of the physics-compensation loss is presented in Appendix B.

### 3.5. Joint Optimization Strategy

The parameter-manifold distillation and physics-compensation mechanisms operate in a coordinated manner to form an end-to-end optimization framework. A joint optimization strategy is adopted, in which a composite loss function simultaneously enforces data-fitting accuracy, distillation consistency, and dynamical consistency.

The total objective function $L_{\mathrm{total}}$ integrates the data-fitting term $L_{\mathrm{data}}$, the output-response distillation term $L_{\mathrm{KD}}$, the parameter-structure alignment terms $L_{\mathrm{Fisher}}$ and $L_{\mathrm{subspace}}$, and the physics-compensation term $L_{\mathrm{phy}}$, as defined in Equation (10):

(10) $$L_{\mathrm{total}}=L_{\mathrm{data}}+\alpha L_{\mathrm{KD}}+\beta L_{\mathrm{Fisher}}+\delta L_{\mathrm{subspace}}+\gamma L_{\mathrm{phy}}$$

The definitions and roles of each loss component are described as follows.

The basic supervision term $L_{\mathrm{data}}$ ensures that the model retains fundamental fitting capability with respect to the ground-truth labels. To mitigate the influence of batch size (*B*) and time-series length (*T*) on gradient magnitude, a normalized formulation is adopted, as defined in Equation (11):

(11) $$L_{\mathrm{data}}=\frac{1}{BT}\sum_{i=1}^{B}\sum_{t=1}^{T}{∥\tau_{\mathrm{GT}}^{\left(i,t\right)}-\tau_{S}^{\left(i,t\right)}∥}_{2}^{2}$$

The output-response distillation term $L_{\mathrm{KD}}$ encourages the student model to inherit the regression behavior of the teacher at the output layer by minimizing the discrepancy between the teacher’s prediction $\tau_{T}$ and the student’s prediction $\tau_{S}$, as defined in Equation (12):

(12) $$L_{\mathrm{KD}}=\frac{1}{BT}\sum_{i=1}^{B}\sum_{t=1}^{T}{∥\tau_{T}^{\left(i,t\right)}-\tau_{S}^{\left(i,t\right)}∥}_{2}^{2}$$

The structural terms $L_{\mathrm{Fisher}}$ and $L_{\mathrm{subspace}}$ implement the parameter-manifold alignment mechanism and facilitate transfer of the teacher’s inference structure, whereas $L_{\mathrm{phy}}$ corresponds to the physics-compensation mechanism described in Section 3.4.

During joint training, the relative gradient contributions of different mechanisms are balanced by weighting coefficients α, β, δ, and γ, which are treated as hyperparameters and tuned independently of the model architecture (selection criteria are provided in Appendix C).

To ensure numerical stability in the multi-objective optimization process, gradient clipping and learning rate warm-up strategies are adopted. These auxiliary strategies suppress early-stage gradient oscillations and promote stable convergence of both physics constraints and parameter-alignment mechanisms. The detailed training procedure and implementation specifics are provided in Appendix C (Algorithm A1).

## 4. Experiments

### 4.1. Experimental Setup and Evaluation Metrics

To evaluate the performance of the proposed framework in lower-limb periodic movement scenarios, Dataset A was used as the primary development dataset [38]. Meanwhile, Dataset B served as an independent testing dataset [39]. Dataset A comprises multimodal recordings from 22 subjects across six activity scenarios: level walking, uphill walking, downhill walking, stair ascent, stair descent, and treadmill locomotion. Dataset B served as an independent cross-dataset validation set to assess the model’s generalizability under varying data-acquisition conditions and subject distributions. Ground-truth joint torques were obtained using a Vicon motion capture system in conjunction with force plates, and computed via OpenSim inverse-dynamics analysis [40].

Data preprocessing was conducted under the assumption of inter-cycle independence. All sequences underwent outlier removal, interpolation, and temporal normalization (resampled to 101 time steps) to conform to the standard input format for periodic gait analysis and to ensure temporal consistency across samples. The experimental task used triaxial IMU acceleration and angular velocity signals within a single gait cycle as inputs to regress joint torque sequences for the hip, knee, and ankle joints. This configuration focuses on lower-limb movements for which the rigid-body dynamics assumption is reasonably valid. The data were partitioned into training, validation, and test sets at a ratio of 7:2:1. Both the teacher and student models were evaluated on identical splits to ensure a fair and unbiased comparison. Data partitioning, parameter initialization, and training randomization were conducted using fixed random seeds to ensure experimental reproducibility.

The evaluation framework assesses PDC-KD model performance across four dimensions, assessing performance across four dimensions: prediction accuracy, peak-error robustness, physical consistency, and computational efficiency. Accuracy metrics include the coefficient of determination ($R^{2}$), root mean square error (RMSE), normalized root mean square error (NRMSE), and Pearson correlation coefficient (PCC) [1,16].

Peak Error quantifies the maximum deviation in predicting critical extrema, reflecting robustness in safety-critical scenarios. In this study, Peak Error is reported as the maximum absolute deviation at the predicted torque extrema. Physical consistency is evaluated using the physical-consistency error (PCE), which is defined as the residual norm of the Newton–Euler consistency equation and quantifies how closely predictions satisfy rigid-body dynamics. Computational efficiency is assessed using the number of trainable parameters, floating-point operations (FLOPs), and inference latency. This evaluation framework emphasizes the effects of structural distillation and physics-compensation mechanisms, providing a quantitative basis for characterizing performance under conditions of representation degradation.

### 4.2. Performance Analysis Under Representation Degradation

The PDC-KD model was evaluated on the primary development dataset (Dataset A) and an independent validation dataset (Dataset B). Table 1 presents representative results for level walking (Dataset A, Walk) and incline locomotion (Dataset B, Incline). Comprehensive results for additional multi-terrain tasks—including ramp ascent and descent, stair negotiation, and treadmill walking—are provided in Appendix D.

#### 4.2.1. Estimation Accuracy and Robustness

As shown in Table 1, for the level-walking task in Dataset A, the student model achieved an $R^{2}$ of 0.949±0.005 and an RMSE of 0.0774±0.003 for the hip joint, corresponding to an NRMSE of 3.04±0.14%. For the knee joint, the $R^{2}$ and NRMSE were 0.776±0.009 and 4.39±0.09%, respectively, whereas for the ankle joint, the corresponding values were 0.891±0.005 and 5.17±0.11%.

In comparison, the teacher model achieved $R^{2}$ values of 0.971, 0.795, and 0.900 for the hip, knee, and ankle joints, respectively, indicating moderate accuracy degradation in the student model. Despite the absence of explicit time–frequency representations and a substantial reduction in model parameters, prediction errors remained within practically acceptable ranges for wearable deployment. Notably, the hip-joint NRMSE increased by only approximately 0.74%, confirming retention of core dynamical feature-extraction capability under edge-computing constraints.

For the incline locomotion task in Dataset B, the student model achieved $R^{2}$ values of 0.965±0.001 and 0.968±0.001 for the hip and knee joints, respectively, compared with 0.963 and 0.970 for the teacher model. Results on the independent validation dataset demonstrate that the parameter-manifold distillation strategy enables the student model to inherit robust structural characteristics across heterogeneous architectures. The physics-guided mechanism further introduces trend-level constraints that enhance cross-condition prediction stability.

#### 4.2.2. Computational Efficiency and Real-Time Feasibility

Table 2 summarizes the quantitative comparison between the teacher and student models in terms of parameter count, computational complexity, and inference efficiency. The teacher model contains 32.24 M parameters and requires 3257.21 M floating-point operations (FLOPs) per forward pass. In contrast, the student model contains only 0.45 M parameters and reduces the computational cost to 45.27 M FLOPs.

In terms of inference efficiency, the teacher model exhibits a latency of 1.97 ms per forward pass (approximately 506 FPS), whereas the student model reduces latency to 1.02 ms (approximately 980 FPS). These results demonstrate that the proposed PDC-KD framework substantially reduces model size and computational complexity while satisfying real-time inference requirements.

#### 4.2.3. Evolution of Physics-Guided Consistency

**Figure 7:**
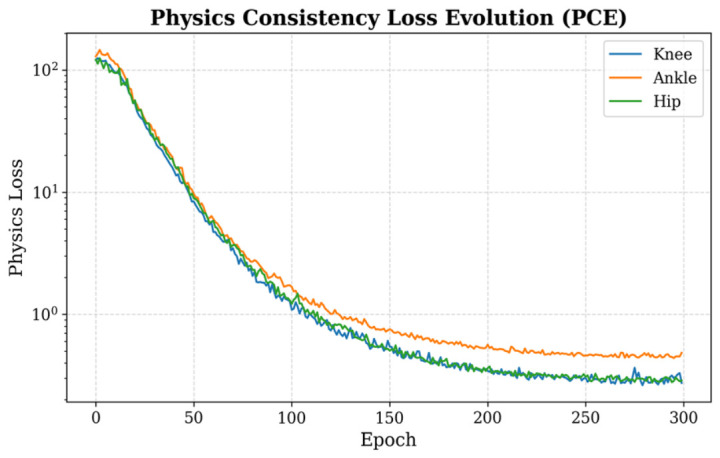
Evolution of physics-consistency error (PCE) during training for the ramp-ascent task in Dataset A. The curves correspond to the hip, knee, and ankle joints.

Figure 7 illustrates the evolution of the physical-consistency error (PCE) across training epochs for the ramp-ascent task in Dataset A. During the initial training phase, PCE values for the hip, knee, and ankle joints are relatively high and exhibit noticeable fluctuations. As training progresses, the PCE for all three joints decreases steadily and gradually stabilizes. By the end of training, the PCE converges to 0.259, 0.403, and 0.633 for the hip, knee, and ankle joints, respectively. The observed convergence of PCE indicates that the physics-compensation mechanism functions as a structural regularizer during optimization, constraining the predicted solution space within the numerical bounds imposed by the dynamic equations.

**Figure 8:**
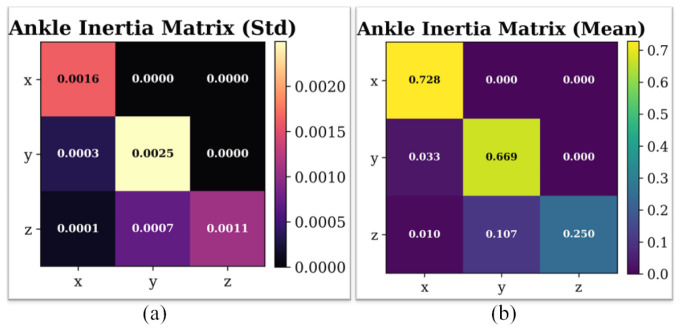
Learned equivalent inertia matrix for the ankle joint in the StairAscent task. (**a**) Mean values across training runs. (**b**) Standard deviation across different random initializations.

Using the ankle joint in the StairAscent task as an example, Figure 8 presents the statistical characteristics of the learned equivalent inertia matrix. Figure 8a illustrates the mean distribution of the inertia-matrix parameters across multiple training runs. The main diagonal elements dominate, whereas the off-diagonal elements exhibit relatively small magnitudes. This distribution pattern indicates that the equivalent inertia parameters function as engineering compensation variables and converge to a numerically stable state during optimization.

Figure 8b presents the corresponding standard-deviation distribution, where each matrix element exhibits low variance across different random initializations. The low variance confirms that the implicit parameterization strategy exhibits strong learnability and convergence consistency. It further indicates that the model does not require explicit calibration of actual biomechanical parameters, but instead adaptively identifies an engineering-optimal solution that satisfies dynamical trend constraints.

#### 4.2.4. Manifold Visualization and Statistical Stability

**Figure 9:**
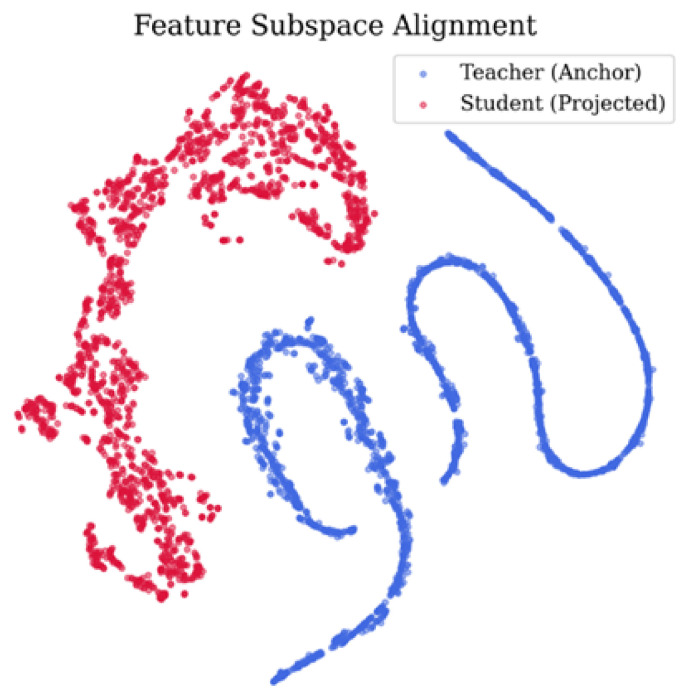
Treadmill t-SNE visualization of anchor-space representations for the ankle joint, comparing teacher (anchor) and student (projected) features.

To investigate the interaction mechanism between heterogeneous architectures in parameter-manifold distillation, Figure 9 presents t-SNE visualizations of the ankle-joint anchor space for the treadmill walking task. The teacher model (blue points), which receives complete time–frequency input, exhibits a continuous and compact manifold structure in the projection space. In contrast, the student model (red points) displays greater dispersion and sparsity.

This discrepancy reflects the design principle of the anchor space, which captures the second-order correlation structure of model parameters and serves as a mediator for distillation computation. Importantly, this mechanism operates independently of direct alignment of input features or semantic representations. Notably, the student model does not replicate the continuous distribution pattern of the teacher model at the numerical level, which is consistent with the intended design objective of the proposed method.

Despite the absence of explicit distribution-alignment constraints, the student model’s projected sample distribution exhibits an overall grouping pattern similar to that of the teacher model. This qualitative observation suggests that parameter-layer distillation influences the parameter-update trajectory, alleviates dependence on feature alignment, and preserves discrimination among different motion states. It should be noted that this visual analysis provides auxiliary insight into training behavior and does not constitute direct evidence of parameter-structure consistency.

**Figure 10:**
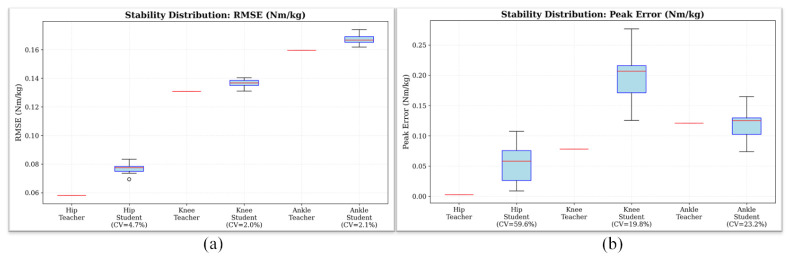
Statistical stability analysis across 10 independent runs for the level-walking task. (**a**) RMSE distribution. (**b**) Peak Error distribution for hip, knee, and ankle joints. The red horizontal line in each box indicates the median value.

Figure 10 illustrates the statistical stability of Dataset A across ten independent experimental repetitions. Under the level-walking experimental setting, prediction errors for each joint exhibit only minor fluctuations across different random initializations. The coefficients of variation (CV) of RMSE for the hip, knee, and ankle joints are 4.7%, 2.0%, and 2.1%, respectively. These results indicate that including the physics-compensation term improves numerical stability during training by suppressing convergence toward locally optimal solutions that violate physical constraints, thereby enhancing the reliability of inference in engineering deployments.

### 4.3. Ablation Study

To evaluate the individual contributions and combined effects of the PDC-KD framework components, an ablation study was conducted on the Walk task using the development dataset (Dataset A). To ensure comparability, all variants employed the same input modality, data partitioning scheme, random seeds, and number of training epochs as the main experiment. Each model variant was trained and evaluated over ten independent runs, and results are reported as mean ± standard deviation (Mean ± Std). Evaluation metrics included $R^{2}$, RMSE, NRMSE, and physical-consistency indicators (PCE and Peak Error).

#### 4.3.1. Effectiveness of the Distillation Strategy

To investigate the roles of parameter-manifold distillation and physics-guided mechanisms in heterogeneous architecture transfer, four progressively enhanced model variants were constructed:

**M0 (Baseline)**: A purely data-driven network trained solely under MSE supervision, without distillation, serving as a lower-bound performance reference.

**M1 (Vanilla KD)**: Introduces standard output-response distillation, aligning only the teacher and student predictions.

**M2 (Geo-KD)**: Extends M1 by incorporating parameter-structure alignment, including Gram-matrix mapping, Fisher-information weighting, and subspace projection.

**M3 (PDC-Full)**: The complete framework, which further integrates the physics-compensation mechanism on top of M2.

The results in Table 3 indicate that, compared with M0, M1 provides only marginal improvement for certain joints (e.g., the hip). Under structural degradation of the input modality (2D to 1D), reliance solely on output-level soft-label alignment is insufficient to transfer the high-dimensional inference logic of the teacher model.

With the introduction of parameter-geometric alignment in M2, the mean $R^{2}$ values for the knee and ankle joints exceed those of M1. This performance improvement empirically supports the assumption that parameter-based structural surrogates constrain the student model’s parameter search space under feature-misalignment conditions, thereby promoting the learning of more robust dynamical representations.

The complete framework (M3) maintains high predictive accuracy (e.g., knee RMSE = 0.136) and substantially reduces physical-consistency violations compared with M0 (hip PCE decreases from 0.279 to 0.256).

#### 4.3.2. Impact of Robust Physics Operators

The role of robust physics operators in ensuring numerical stability is further evaluated by comparison with a naïve physics-constraint formulation. In the naïve setting, inertia parameters are learned as unconstrained variables without enforcing symmetric positive-definite (SPD) constraints. In contrast, the robust formulation (ours) employs Cholesky-based reparameterization of the inertia matrix to guarantee algebraic validity. To eliminate confounding factors in signal processing, both configurations apply the same Savitzky–Golay filter.

As shown in Table 4, Peak Error values for all joints under the robust formulation are lower than those under the naïve formulation (e.g., the ankle Peak Error decreases from 0.235 to 0.230). These results indicate that enforcing symmetric positive-definite (SPD) constraints effectively narrows the feasible solution space and mitigates physically implausible peak predictions near extrema.

In the level-walking task, both formulations exhibit high numerical stability (CV < 1%). Although the average PCE for certain joints (e.g., knee and ankle) is slightly higher under the robust formulation, this reflects an inherent trade-off between data fitting and physical validity. The naïve formulation permits parameters to explore physically invalid regions (e.g., non-positive-definite matrices) to overfit noisy data, thereby achieving lower numerical residuals at the expense of physical interpretability. In contrast, the robust strategy prioritizes the algebraic validity of inertial parameters. Although numerical fitting is subject to stronger constraints, this design better prevents divergence toward physically invalid solutions, thereby enhancing engineering reliability under out-of-distribution conditions.

The ablation results indicate that parameter-manifold distillation primarily enhances data-fitting performance, whereas the physics-compensation mechanism establishes structural boundaries within the constrained parameter space. Together, the two mechanisms play complementary roles in structural knowledge transfer and physical-consistency enforcement.

### 4.4. Comparison with Baseline Methods and Discussion

#### 4.4.1. Baseline Setup

This section compares the performance of various knowledge-transfer and structural-adaptation strategies under heterogeneous representation conditions, focusing on their impact on model accuracy and stability. No new methodologies are introduced, and hyperparameters remain consistent across all baselines. Experiments follow the standard evaluation scenario of the level-walking task in Dataset A (development set), assessing model performance in conventional gait-dynamics modeling.

To ensure fairness and reproducibility, all baseline experiments follow the same training and evaluation protocol as the main study. The protocol includes identical data partitioning, fixed random seeds, 200 training epochs, and the AdamW optimization algorithm. Each model is independently trained and evaluated five times under identical settings, and results are reported as mean ± standard deviation (Mean ± Std) to assess statistical stability. Evaluation metrics include prediction accuracy ($R^{2}$ and RMSE) and physical-safety indicators (PCE and Peak Error), enabling quantitative comparison of different strategies in suppressing physically inconsistent prediction biases.

#### 4.4.2. Baseline Model Definitions

To validate the theoretical analysis of the heterogeneous gap, baseline models representing different technical paradigms were selected. The classification criteria and selection rationale are summarized in Table 5.

The teacher model serves as a high-precision reference, leveraging high-information-density time–frequency inputs that implicitly embed physical dynamics. The student (no KD) model represents the absence of knowledge transfer mechanisms, reflecting the baseline performance achievable with raw IMU signals alone. The larger student model evaluates the effect of increasing model capacity without introducing structural or physical constraints. FitNets represents a typical feature-level distillation approach, introducing adaptation layers to align heterogeneous feature representations. In contrast, PDC-KD relies on structural stability in parameter space and incorporates physical laws as an external compensation mechanism.

#### 4.4.3. Quantitative Results and Analysis

Table 6 presents the quantitative comparison results for the hip joint in the level-walking task of Dataset A; the hip joint is selected as a representative case due to its dominant role in lower-limb locomotion dynamics.

The results indicate that the larger student yields only marginal improvements in $R^{2}$ and RMSE compared with the baseline student (no KD) ($R^{2}\approx 0.959$ for Student (No KD) vs. 0.955 for Larger Student), while the Peak Error remains high (0.4418). This suggests that, under the current experimental setting, the primary performance bottleneck of the lightweight model arises from structural loss of observability at the input stage (i.e., loss of frequency-domain information), rather than from limitations in parameter capacity. Under degraded observational conditions, increasing network depth or width alone is insufficient to reconstruct the missing dynamical mapping relationships.

The feature-level distillation approach (FitNets) demonstrates limited effectiveness ($R^{2}=0.9536$). These findings are consistent with prior analyses: when the input modality degrades 2D time–frequency spectra to 1D time-series signals, significant heterogeneity prevents intermediate feature layers from satisfying the manifold-isomorphism assumption. Consequently, linear projection layers cannot effectively bridge the order-of-magnitude difference in information density, resulting in only marginal accuracy gains from forced feature alignment.

In contrast, the PDC-KD framework demonstrates more balanced performance under engineering degradation conditions. Although the $R^{2}$ value (0.9590) is comparable to that of the purely data-driven model, physical-safety performance improves substantially, with Peak Error reduced by approximately 63% (from 0.4569 to 0.169). This result indicates that, by avoiding unstable intermediate feature alignment and instead leveraging parameter-manifold inheritance combined with physics-guided compensation, the framework establishes a feasible pathway for maintaining prediction reliability under incomplete representation conditions. Overall, the experimental evidence confirms that, for lightweight dynamic estimation under edge-computing constraints, the synergy between structured knowledge carriers and physics-consistency compensation provides a more robust engineering solution than capacity expansion or feature-level alignment alone.

## 5. Discussion

The experimental results indicate that degrading the input modality from an explicit two-dimensional time–frequency representation to a one-dimensional time-series signal does not lead to a complete collapse in task performance. Although geometric alignment between teacher and student feature distributions is weakened, the student model retains the capacity to perform the core dynamical regression task under engineering constraints. This observation suggests that, under structural reduction of input dimensionality, the suitability of feature alignment as a primary knowledge carrier must be carefully reconsidered [10]. Previous approaches, such as FitNets and Relational KD, are primarily predicated on the assumption that the observation spaces of teacher and student models are isomorphic or only weakly heterogeneous. However, under the two-dimensional-to-one-dimensional degradation setting examined in this study, the shared mapping basis in feature space is substantially weakened, thereby reducing observability. Nevertheless, the results demonstrate that even in the absence of fine-grained feature geometry from the teacher model, task-relevant information can still be transferred across heterogeneous architectures.

Experimental observations and ablation analyses indicate that parametric geometric constraints do not require the student model to reconstruct the teacher’s intermediate feature representations. As illustrated in Figure 9, after introducing parameter-geometric constraints, the anchor-space projections of the student model remain relatively discrete and do not converge toward the teacher manifold. Table 3 further demonstrates that M2 improves performance for multiple joints, particularly the knee and ankle, compared with M1. These findings suggest that, under heterogeneous distillation settings, parameter-geometric constraints primarily influence the optimization trajectory and the model’s discriminative structure [42].

This effect may be related to the intrinsic biomechanical coordination of human movement. Although IMU signals are high-dimensional and contain substantial noise, lower-limb motion is inherently constrained by skeletal structure and muscle coordination [43]. The principal task-related directions in the teacher’s parameter space are therefore more likely to encode genuine dynamical patterns rather than high-dimensional noise-induced redundancy. Consequently, even without reconstructing the teacher’s two-dimensional time–frequency manifold at the feature level, the student model can inherit essential structural information by enforcing parametric-geometric consistency (e.g., Gram-structure or principal-subspace alignment).

This structural-surrogate mechanism provides a plausible explanation for the non-uniform performance observed across joints. As shown in Table 1 and Table 3, distillation gains are relatively stable for the hip joint. In contrast, improvements for the knee joint—characterized by more substantial impact and more complex transient dynamics—are more limited. In scenarios involving pronounced transient dynamics, the effect of purely structural constraints may diminish. This observation suggests that structural consistency alone may be insufficient to compensate fully for observability degradation, thereby motivating the incorporation of additional physics-guided constraints [44].

Under conditions of incomplete observability, the primary contribution of the physics-guided mechanism is to enhance predictive stability. Specifically, it suppresses high-frequency anomalous oscillations induced by input dimensionality reduction, constrains predictions within physically plausible ranges, and provides trend-level constraints derived from dynamic equations when fine-grained spectral information is unavailable due to input dimensionality reduction. From an optimization perspective, the physical-consistency loss influences the direction of parameter updates. Compared with training based solely on label supervision, this mechanism imposes additional penalties on update directions that violate dynamic constraints, thereby restricting the model’s feasible solution space [45]. In this study, physical constraints are embedded directly into the training loss function rather than implemented as post hoc correction modules. Experimental results show that reductions in physical-consistency error (PCE) and Peak Error are more pronounced than reductions in average error (RMSE), consistent with improved control over the tails of the error distribution. However, this mechanism cannot fully compensate for information loss induced by representation degradation, and its effectiveness diminishes under conditions dominated by high-frequency shocks or strong nonlinear dynamics.

It is also important to clarify the dynamical applicability boundaries of the physics-guided compensation mechanism. The Newton–Euler formulation adopted here assumes rigid-body kinematics, which provides a reasonable approximation for periodic lower-limb locomotion scenarios. However, for strongly nonlinear or aperiodic motions, such as rapid turning, jumping, or sudden posture changes, the rigid-body assumption may no longer hold. In such cases, soft-tissue deformation and multi-segment coupling effects may cause the physics-compensation term to introduce systematic bias rather than beneficial correction. The effectiveness of the proposed mechanism is therefore expected to diminish as the target motion deviates from the periodic locomotion conditions for which the framework was designed. Future work could explore incorporating musculoskeletal or flexible-body models to broaden applicability beyond the current rigid-body constraint.

Although this study validates the effectiveness of the proposed method under specific experimental conditions, several limitations must be clarified to define its scope of applicability. First, there is an inherent trade-off between training cost and online efficiency. Although the PDC-KD framework substantially reduces online inference latency (1.02 ms), it introduces additional computational overhead during training. Specifically, anchor-space Fisher information estimation, singular value decomposition (SVD), and Cholesky-based reparameterization of physical parameters increase the complexity of offline training. This reflects a deliberate offline-for-online computational trade-off; however, its suitability for scenarios with frequent retraining or edge-side adaptive learning requires further investigation. To quantify the offline training overhead introduced by the PDC-KD framework, wall-clock timing was recorded for the stair-ascent task. Fisher information precomputation required approximately 0.47 s per joint, and SVD decomposition of the anchor-space Gram matrix required less than 0.1 s. Each student training run (200 epochs) took approximately 166 s per joint, with a peak GPU memory consumption of 891 MB. The detailed breakdown is provided in Appendix D (Table A3). These results confirm that the additional offline overhead is incurred only once prior to deployment and does not affect online inference latency (1.02 ms, approximately 980 FPS).

Second, the engineering equivalence of the learned physical parameters warrants careful consideration. Obtained through implicit optimization during training, the equivalent inertia tensor represents an engineering surrogate rather than an exact biomechanical parameter. It constrains the numerical trend and feasible region of predictions but does not provide a precise physiological interpretation. Its physical consistency and stability under abnormal movements or extreme operating conditions remain to be systematically validated. Furthermore, the current evaluation is limited to open-loop, offline testing within standard gait cycles, and real-time stability in closed-loop control systems has not yet been verified. Future work will explore more efficient adaptive mechanisms to accommodate evolving teacher models and non-stationary data distributions [25].

To assess the robustness of the framework under sensor measurement uncertainty, a post hoc sensitivity analysis was performed by injecting additive Gaussian noise (σ∈{0.0,0.1,0.2,0.5,1.0} relative to the normalized input scale) into the student model’s IMU inputs at inference time. As reported in Table A4, RMSE degradation remained below 7% for σ≤0.2 across all three joints, indicating adequate robustness under realistic sensor noise levels. The physical-consistency error exhibited negligible variation (<1%) across all noise conditions, suggesting that the physics-guided compensation mechanism imposes structural constraints that are largely invariant to input-level perturbations. Performance degrades substantially at σ=1.0, consistent with the expectation that the framework targets realistic rather than adversarial noise conditions.

Third, several sources of uncertainty in both input data and model parameters warrant acknowledgment. On the input side, IMU signals are subject to soft-tissue artifacts, sensor-to-segment misalignment, and measurement drift over prolonged use, which introduce uncertainty into the observed kinematics. On the model side, the equivalent inertia tensor $I_{\mathrm{eff}}$ functions as an implicitly learned engineering surrogate whose convergence behavior may vary across subjects with substantially different body segment parameters. Furthermore, the Fisher information matrix is precomputed on the training distribution and does not adapt to inter-subject variability or domain shift. These factors suggest that the reported performance metrics reflect aggregate behavior under the evaluated experimental conditions and may not generalize uniformly to all individual deployment scenarios.

A further practical consideration concerns the gap between the offline evaluation protocol and real-world wearable deployment. Experimental evaluation in this work relies on pre-segmented, time-normalized gait cycles, under the implicit assumption that gait-phase segmentation has already been performed upstream. In continuous wearable deployment, the model must process an uninterrupted IMU data stream in which gait-cycle boundaries are not known a priori. Integrating the proposed framework with a real-time segmentation front-end—such as a heel-strike detector or a sliding-window gait-phase classifier—would likely be necessary for practical deployment. However, this integration has not been systematically evaluated, and assessment on continuous, non-segmented sequences under realistic ambulatory conditions constitutes an important direction for future work. Accordingly, the present results should be interpreted within the scope of cycle-level offline evaluation.

Finally, the generalizability of the Fisher information weighting strategy warrants further consideration. In the current framework, the Fisher information matrix $F_{T}$ is precomputed from the teacher model on the training distribution and remains fixed during student training. When the target data distribution shifts substantially—due to changes in subject demographics, activity type, or sensor configuration—the precomputed weights may no longer accurately reflect task-relevant parameter sensitivity, potentially limiting distillation effectiveness. A possible mitigation could involve periodically re-estimating $F_{T}$ on a representative subset of the target distribution; alternatively, an incremental updating scheme could be adopted. These extensions are left for future work. As noted in Section 4.2, the t-SNE visualization serves as qualitative evidence rather than causal proof.

Despite these limitations, the findings provide practical insights for designing resource-constrained edge-learning systems. In scenarios involving simplified input modalities and constrained computational budgets, incorporating parameter-space structural constraints and physics-consistency mechanisms can enhance prediction stability while preserving model compactness. More broadly, integrating structural consistency with physical rationality facilitates a balanced trade-off between system efficiency and stability in similar tasks.

## 6. Conclusions

To address representation degradation arising from the transition of two-dimensional time–frequency spectra to one-dimensional time-series signals under edge-computing constraints, this study proposes a Physically Guided Dual-Consistency Knowledge Distillation (PDC-KD) framework. The framework integrates parameter-manifold distillation with exogenous physics-guided compensation to mitigate accuracy degradation and loss of physical consistency in lightweight models under reduced observability. Experimental evaluation on a standard lower-limb gait dataset demonstrates that PDC-KD reduces the parameter count by 98% while achieving low-latency inference (1.02 ms). The resulting student model maintains prediction accuracy comparable to that of a high-fidelity teacher model and significantly reduces physical-consistency error and Peak Error, thereby validating its engineering reliability and effectiveness. Although the current validation is limited to lower-limb periodic gait scenarios under rigid-body dynamic assumptions, future work will investigate the robustness of physics-guided compensation in complex discrete motion tasks and further optimize deployment for ultra-low-power embedded platforms. Overall, this work demonstrates a viable technical pathway for wearable dynamics estimation under constrained engineering resources, achieving a balance among computational efficiency, predictive accuracy, and physical reliability.

## Figures and Tables

**Table 1:** Comparison of prediction accuracy (mean ± standard deviation) between the Teacher (T) and Student (S) models for representative tasks.

Dataset	Joint	$R^{2}$ (T)	$R^{2}$ (S)	RMSE (T)	RMSE (S)	NRMSE (T)	NRMSE (S)
Dataset A (Walk)	Hip	0.971	0.949±0.005	0.0629	0.0774±0.003	2.30	3.04±0.14
	Knee	0.795	0.776±0.009	0.1328	0.1356±0.003	4.20	4.39±0.09
	Ankle	0.900	0.891±0.005	0.1549	0.1666±0.006	4.94	5.17±0.11
Dataset B (Incline)	Hip	0.963	0.965±0.001	0.1195	0.1490±0.016	3.40	3.30±0.04
	Knee	0.970	0.968±0.001	0.0727	0.1200±0.024	2.39	2.48±0.03

**Table 2:** Comparison of computational efficiency between the teacher and student models.

Model	Parameters (M)	FLOPs (M)	Latency (ms)	FPS
Teacher	32.24	3257.21	1.97	506
Student (Ours)	0.45	45.27	1.02	980

**Table 3:** Ablation study on different distillation strategies (mean ± standard deviation). Bold values indicate the best performance among M0–M3.

Method	Joint	$R^{2}$	RMSE	NRMSE (%)	PCE
M0: Baseline	Hip	0.9588±0.0014	0.069±0.001	2.73±0.05	0.279±0.001
	Knee	0.7659±0.0114	0.140±0.003	4.49±0.11	0.222±0.002
	Ankle	0.8830±0.0070	0.173±0.005	5.34±0.16	0.399±0.003
M1: Vanilla KD	Hip	0.9582±0.0019	0.069±0.002	2.75±0.06	0.269±0.001
	Knee	0.7698±0.0034	0.139±0.001	4.45±0.03	0.223±0.001
	Ankle	0.8823±0.0030	0.173±0.002	5.36±0.07	0.395±0.002
M2: Geo-KD	Hip	0.9583±0.0022	0.069±0.002	2.75±0.07	0.269±0.001
	Knee	0.7685±0.0033	0.139±0.001	4.46±0.03	0.223±0.002
	Ankle	0.8855±0.0012	0.171±0.001	5.29±0.03	0.396±0.002
M3: PDC-Full (Ours)	Hip	0.9588±0.0013	0.069±0.001	2.73±0.04	0.256±0.001
	Knee	0.7784±0.0033	0.136±0.001	4.37±0.03	0.406±0.003
	Ankle	0.8857±0.0042	0.171±0.003	5.28±0.10	0.625±0.003

**Table 4:** Ablation on physics constraint formulations.

Physics Design	Joint	PCE	Peak Error	Stability (CV)
Naïve Physics	Hip	0.269±0.001	0.171	0.25%
	Knee	0.223±0.002	0.208	0.75%
	Ankle	0.396±0.002	0.235	0.39%
Robust Physics (Ours)	Hip	0.256±0.001	0.169	0.40%
	Knee	0.406±0.003	0.203	0.84%
	Ankle	0.625±0.003	0.230	0.51%

**Table 5:** Classification and technical characteristics of baseline models.

Method	Strategy Category	Core Technical Implementation	Physical Awareness
Teacher	Upper-Bound Reference	Based on 2D time–frequency representations and a deep ResNet architecture	No (Implicit Physical Modeling)
Student (No KD)	Lower-Bound Reference	Purely data-driven lightweight RNN (M0), trained solely under label supervision	No
Larger Student	Capacity Expansion Strategy	Expands recurrent network capacity, increasing parameter size to 0.62 M	No
FitNets [41]	Feature-Level Alignment	Establishes alignment in intermediate feature space via linear projection layers	No
PDC-KD (Ours)	Structural Collaboration Strategy	Integrates parameter-manifold inheritance and physics-guided robust compensation mechanisms	Yes

**Table 6:** Performance comparison of different strategies in the level-walking task (hip joint). Bold values indicate the best performance among all compared methods.

Method	$R^{2}$	RMSE	PCE	Peak Error
Teacher	0.971	0.0629	–	–
Student (No KD)	0.9589±0.0020	0.0688	0.279±0.001	0.4569
Larger Student	0.9548±0.0029	0.0721	0.285±0.003	0.4418
FitNets	0.9536±0.0018	0.0731	0.269±0.001	0.4377
PDC-KD (Ours)	0.9590±0.0013	**0.0688**	0.256±0.001	**0.169**

## Data Availability

The source code (v1.0) supporting the conclusions of this study is publicly available on GitHub at https://github.com/Hihubxu/PDC-KD (accessed on 15 February 2026). Dataset A is hosted by the EPIC repository at Georgia Tech and is accessible at https://www.epic.gatech.edu/opensource-biomechanics-camargo-et-al/ (accessed on 15 February 2026). Dataset B corresponds to “A Human Lower-Limb Biomechanics and Wearable Sensors Dataset During Cyclic and Non-Cyclic Activities” and is available from the Georgia Tech Repository at https://repository.gatech.edu/entities/publication/20860ffb-71fd-4049-a033-cd0ff308339e/ (accessed on 15 February 2026).

## Appendix A. Implementation of Robust Dynamics Operators

This section details the computational procedures of the dynamic operators introduced in Section 3.4 of the main text.

### Appendix A.1. Cholesky Parameterization of the Inertia Tensor

To enforce symmetric positive-definite (SPD) algebraic constraints on the equivalent inertia tensor $I_{\mathrm{eff}}$ during optimization, the tensor is reparameterized via Cholesky decomposition:

(A1) $$I_{\mathrm{eff}}=LL^{⊤},\;L\in R^{3\times 3}$$

where *L* is a learnable lower-triangular matrix. The lower-triangular structure implicitly constrains parameter updates, ensuring that the resulting inertia tensor remains within the feasible SPD domain during numerical optimization.

### Appendix A.2. Angular Acceleration Estimation Procedure

For the input angular-velocity time series ω, a Savitzky–Golay (SG) filter is applied to compute a smoothed first derivative, providing an approximate estimate of angular acceleration:

(A2) $${\dot{\omega }}_{\mathrm{filt}},\;\omega_{\mathrm{filt}}=\mathrm{SGFilter}\left(\omega_{\mathrm{raw}}\right)$$

The filtered angular velocity $\omega_{\mathrm{filt}}$ and its derivative ${\dot{\omega }}_{\mathrm{filt}}$ are subsequently substituted into the dynamic equations as stabilized inputs. The specific window length and polynomial order of the SG filter are provided in Appendix C.2.

## Appendix B. Formulation of Physics Consistency Loss

This section provides the detailed computational formulation of Equation (9) in the main text.

### Appendix B.1. Definition of Physical Residual

Based on Equation (8), the physical residual is defined as the Euclidean distance between the student network output $\tau_{S}$ and the dynamically computed torque $\tau_{\mathrm{dyn}}$. The physics-consistency loss $L_{\mathrm{phy}}$ is formulated as:

(A3) $$L_{\mathrm{phy}}=\frac{1}{BT}\sum_{i=1}^{B}\sum_{t=1}^{T}{∥\tau_{S}^{\left(i,t\right)}-\tau_{\mathrm{dyn}}^{\left(i,t\right)}∥}_{2}^{2}$$

where *B* denotes the batch size, *T* denotes the sequence length (number of time steps per sample), and $\tau_{\mathrm{dyn}}$ is calculated using the dynamic operators defined in Appendix A and the current equivalent inertia tensor $I_{\mathrm{eff}}$. During backpropagation, gradients of this loss are used to update both the student network parameters $\theta_{S}$ and the Cholesky factor *L*.

### Appendix B.2. Optimization Characteristics of $L_{\mathrm{phy}}$

During joint optimization, $L_{\mathrm{phy}}$ functions as a structural regularization term:

1. **Cold-start phase:** At early training stages, $I_{\mathrm{eff}}$ has not yet converged, and the influence of physical constraints remains relatively weak. Optimization is therefore primarily driven by the data-fitting loss.
2. **Convergence phase:** As $I_{\mathrm{eff}}$ gradually adapts to subject-specific motion characteristics, $L_{\mathrm{phy}}$ exerts more substantial regularization effects, guiding parameter updates within the feasible region defined by engineering assumptions.

## Appendix C. Training Algorithm and Hyperparameters

### Appendix C.1. Optimization Procedure

Training adopts an end-to-end joint optimization strategy. The teacher model parameters remain frozen, while the student parameters $\theta_{S}$ and learnable Cholesky factors *L* are updated synchronously. The training procedure is summarized in Algorithm A1.

**Algorithm A1** PDC-KD Training Procedure
1: **Input:** Dataset *D*, teacher model *T*, student model *S* 2: Initialize student parameters $\theta_{S}$; set Cholesky factor L=I 3: Load precomputed Teacher Fisher matrix $F_{T}$ and projection operator $P_{T}$ 4: **for** epoch =1 to 200 **do** 5: **if** epoch <10 **then** 6: Apply linear warm-up 7: **end if** 8: **for** batch (x,y)∈D **do** 9: Compute teacher output $\tau_{T}=T\left(x_{\mathrm{cwt}}\right)$ 10: Compute student output $\tau_{S}=S\left(x_{\mathrm{raw}}\right)$ 11: Apply SG filtering to obtain $\left({\dot{\omega }}_{\mathrm{filt}},\omega_{\mathrm{filt}}\right)$ 12: Compute equivalent inertia tensor $I_{\mathrm{eff}}=LL^{⊤}$ 13: Compute $L_{\mathrm{data}},L_{\mathrm{KD}},L_{\mathrm{Fisher}},L_{\mathrm{subspace}},L_{\mathrm{phy}}$ 14: Aggregate total loss $L_{\mathrm{total}}$ (Equation (10)) 15: Perform backpropagation with gradient clipping to update $\theta_{S}$ and *L* 16: **end for** 17: **end for** 18: **Output:** Trained student model *S*

### Appendix C.2. Hyperparameter Configuration

Based on validation-set tuning, the loss weights are configured as follows: distillation weight α=1.0, Fisher weight β=0.01, subspace-alignment weight δ=0.005, and physics-compensation weight γ=0.1. The optimizer is AdamW with an initial learning rate of $1\times 10^{-3}$. The gradient clipping threshold is set to 1.0. The Savitzky–Golay filter window length is 11 with a polynomial order of 3.

### Appendix C.3. Initialization Strategy

The equivalent inertia parameter *L* is initialized as an identity matrix to ensure a physically valid starting point for optimization.

## Appendix D. Supplementary Experimental Results

This appendix provides quantitative results for additional motion conditions not presented in Section 4.2 of the main text. All experiments follow the identical evaluation protocol, dataset partitioning strategy, and random-seed configuration described in the main text.

**Task Definitions:** RA/RD: Ramp ascent/Ramp descent; SA/SD: Stair ascent/Stair descent; TM: Treadmill walking; Walk: Normal walking; Incline: Cross-dataset validation for ramp ascent (Dataset B); Normal: Cross-dataset validation for normal walking (Dataset B).

**Evaluation Metrics:**$R^{2}$, RMSE, NRMSE, and PCC are used to quantify predictive accuracy and numerical fit.

**Statistical Reporting:** Results are reported as mean ± standard deviation across 10 independent runs (N=10). These results are summarized in Table A1 and Table A2. Table A3 reports the offline training overhead of the PDC-KD framework measured on the stair-ascent task. Table A4 presents the noise sensitivity analysis of the student model under varying levels of injected Gaussian noise.

**Table A1:** Prediction accuracy comparison between teacher (T) and student (S) models across multiple locomotion conditions (mean ± standard deviation, N=10).

Task	Joint	$R^{2}$		RMSE		NRMSE (%)		PCC (S)
		T	S	T	S	T	S	
RA	Hip	0.918	0.873±0.026	0.143	0.177±0.018	5.58	6.93±0.70	0.936±0.012
	Knee	0.928	0.831±0.046	0.109	0.165±0.023	4.19	6.34±0.84	0.918±0.021
	Ankle	0.950	0.933±0.009	0.162	0.188±0.014	5.85	6.80±0.43	0.966±0.005
RD	Hip	0.951	0.934±0.002	0.094	0.109±0.006	3.48	4.02±0.06	0.967±0.001
	Knee	0.951	0.947±0.003	0.147	0.153±0.009	5.11	5.31±0.12	0.973±0.001
	Ankle	0.943	0.945±0.004	0.126	0.123±0.010	3.75	3.66±0.14	0.973±0.002
SA	Hip	0.969	0.952±0.002	0.089	0.110±0.005	3.28	4.06±0.07	0.976±0.001
	Knee	0.962	0.958±0.002	0.094	0.098±0.006	3.31	3.46±0.10	0.980±0.001
	Ankle	0.973	0.973±0.001	0.154	0.154±0.005	4.04	4.04±0.06	0.986±0.001
SD	Hip	0.921	0.894±0.010	0.096	0.110±0.010	3.52	4.06±0.18	0.946±0.005
	Knee	0.967	0.962±0.001	0.135	0.145±0.006	4.15	4.44±0.08	0.981±0.001
	Ankle	0.948	0.952±0.002	0.177	0.171±0.007	5.37	5.19±0.11	0.976±0.001
TM	Hip	0.985	0.986±0.000	0.039	0.038±0.001	1.45	1.41±0.02	0.993±0.000
	Knee	0.977	0.981±0.000	0.055	0.049±0.001	1.92	1.72±0.02	0.991±0.000
	Ankle	0.983	0.976±0.006	0.109	0.127±0.020	3.21	3.74±0.57	0.988±0.003
Normal	Hip	0.987	0.987±0.000	0.051	0.050±0.001	1.85	1.84±0.03	0.995±0.001
	Knee	0.989	0.988±0.000	0.039	0.041±0.001	1.35	1.42±0.02	0.994±0.001

**Table A2:** Physics consistency and Peak Error statistics of the student model under multi-terrain locomotion conditions (mean ± standard deviation, N=10).

Task	Joint	PCE	Peak Error
RA	Hip	0.388±0.005	0.121±0.023
	Knee	0.608±0.005	0.069±0.032
	Ankle	0.780±0.005	0.087±0.022
RD	Hip	0.303±0.002	0.035±0.029
	Knee	0.839±0.002	0.020±0.015
	Ankle	0.691±0.003	0.100±0.040
SA	Hip	0.330±0.001	0.067±0.015
	Knee	0.490±0.004	0.061±0.026
	Ankle	0.721±0.003	0.042±0.019
SD	Hip	0.175±0.002	0.060±0.036
	Knee	0.698±0.003	0.161±0.012
	Ankle	0.701±0.003	0.015±0.007
TM	Hip	0.309±0.000	0.100±0.019
	Knee	0.223±0.000	0.033±0.012
	Ankle	0.466±0.016	0.073±0.035
Walk	Hip	0.259±0.001	0.055±0.033
	Knee	0.403±0.004	0.200±0.040
	Ankle	0.633±0.005	0.119±0.028
Incline	Hip	0.297±0.001	0.132±0.026
	Knee	0.275±0.001	0.233±0.029
Normal	Hip	0.426±0.001	0.225±0.017
	Knee	0.329±0.000	0.280±0.037

**Table A3:** Offline training overhead of the PDC-KD framework (stair-ascent task, averaged over three joints and ten independent runs).

Component	Time/Cost
Fisher matrix precomputation	0.47±0.02 s per joint
SVD decomposition	0.05±0.05 s per joint
Student training (200 epochs, 1 run)	166.1±1.6 s per joint
Total offline time (10 runs, 3 joints)	≈83 min
Peak GPU memory	767.5 MB (max 891 MB)
Online inference latency	1.02 ms (≈980 FPS)

**Table A4:** Noise sensitivity analysis of the student model (ramp-ascent task). RMSE and PCE are reported as a function of injected Gaussian noise standard deviation σ.

σ	Hip		Knee		Ankle	
	RMSE	PCE	RMSE	PCE	RMSE	PCE
0.0	0.163	1.026	0.101	5.822	0.127	7.912
0.1	0.165	1.026	0.104	5.822	0.127	7.912
0.2	0.169	1.025	0.108	5.823	0.128	7.912
0.5	0.179	1.020	0.132	5.821	0.163	7.912
1.0	0.255	0.991	0.181	5.818	0.201	7.911
